# Bend or break: how biochemically versatile molecules enable metabolic division of labor in clonal microbial communities

**DOI:** 10.1093/genetics/iyab109

**Published:** 2021-10-01

**Authors:** Sriram Varahan, Sunil Laxman

**Affiliations:** Institute for Stem Cell Science and Regenerative Medicine (inStem), Bengaluru 560065, India

**Keywords:** phenotypic heterogeneity, cross-feeding systems, gluconeogenesis, glycolysis, metabolic specialization, division of labor

## Abstract

In fluctuating nutrient environments, isogenic microbial cells transition into “multicellular” communities composed of phenotypically heterogeneous cells, showing functional specialization. In fungi (such as budding yeast), phenotypic heterogeneity is often described in the context of cells switching between different morphotypes (*e.g.*, yeast to hyphae/pseudohyphae or white/opaque transitions in *Candida albicans*). However, more fundamental forms of metabolic heterogeneity are seen in clonal *Saccharomyces cerevisiae* communities growing in nutrient-limited conditions. Cells within such communities exhibit contrasting, specialized metabolic states, and are arranged in distinct, spatially organized groups. In this study, we explain how such an organization can stem from self-organizing biochemical reactions that depend on special metabolites. These metabolites exhibit plasticity in function, wherein the same metabolites are metabolized and utilized for distinct purposes by different cells. This in turn allows cell groups to function as specialized, interdependent cross-feeding systems which support distinct metabolic processes. Exemplifying a system where cells exhibit either gluconeogenic or glycolytic states, we highlight how available metabolites can drive favored biochemical pathways to produce new, limiting resources. These new resources can themselves be consumed or utilized distinctly by cells in different metabolic states. This thereby enables cell groups to sustain contrasting, even apparently impossible metabolic states with stable transcriptional and metabolic signatures for a given environment, and divide labor in order to increase community fitness or survival. We speculate on possible evolutionary implications of such metabolic specialization and division of labor in isogenic microbial communities.

## Introduction

Unicellular organisms seldom naturally exist as individuals. Rather, they live in communities with varying degrees of complexity (Stahl *et al.* 2006; Callieri *et al.* 2018). This community lifestyle provides many advantages to the individuals within, including enhanced growth/proliferation, better survival in fluctuating environments, and resilience against invaders/cheaters (Shade *et al.* 2012; Dos Santos *et al.* 2018). Microbial communities can be comprised of individuals from (a) different domains of life (lichens), (b) different species within the same kingdom of life (polymicrobial bacterial/fungal communities), or (c) arise from the clonal expansion of a single cell. In all these communities, there exists a diversity among the individuals due to their genetic makeup or variations in the environment they live in (Elowitz *et al.* 2002;Raj and van Oudenaarden 2008;Balázsi *et al.* 2011;Ackermann 2013, 2015). Interestingly, even communities formed by genetically identical cells growing in the same microenvironment, can exhibit substantial phenotypic variation with respect to gene expression patterns, metabolic states, physical properties, response to nutrients and external stresses (Stockholm *et al.* 2007; Ackermann 2015; Takhaveev and Heinemann 2018). Collectively, cellular phenotypic heterogeneity includes all these variations among the individual cells of the community, and this functionally impacts the overall fate of the community (Ackermann 2013, 2015). An unanswered question in the field of microbial phenotypic heterogeneity is, how do genetically identical cells/clonal cells achieve phenotypic heterogeneity within communities, and what does that imply? Here, we highlight emerging ideas that address these questions, focusing primarily on metabolic heterogeneity. We illustrate how given biochemical processes lead to metabolic constraints that enable distinct phenotypic states to sustain themselves within a clonal community.

### What might phenotypic heterogeneity mean to a clonal community?

Phenotypic heterogeneity in clonal cell populations can confer multiple benefits to the individuals of the community. Variations amongst individuals ensure that a subset of the population survives sudden changes in the environment and can thereby allow genotypes to persist in fluctuating conditions. This collective bet-hedging increases the survivability of individuals in the community (López-Maury *et al.* 2008; Beaumont *et al.* 2009; Grimbergen *et al.* 2015). Phenotypic heterogeneity also can lead to division of labor between individuals of the community, wherein different individual cells perform distinct functions. This can enable challenging biochemical processes to operate (van Gestel *et al.* 2015; West and Cooper 2016; Giri *et al.* 2019). Much like other microbial species discussed in other reviews (Süel *et al.* 2007; Stewart *et al.* 2011), several fungi exhibit incredible heterogeneity within clonal communities (Hogan and Gladfelter 2015; Hewitt *et al.* 2016). Well-studied examples include the filamentous fungi *Aspergillus niger* which exhibits heterogeneity at multiple levels: differences between individual spores (*e.g.*, during spore formation, quiescence, or germination), differences between individual hyphae of the same mycelium, or phenotypic variation between distinct, isogenic mycelia (Hewitt *et al*. 2016; Wosten *et al.* 1991; Vinck *et al.* 2005, 2011; de Bekker *et al.* 2011a, b).

### Phenotypic heterogeneity in yeasts

Yeasts are a well-studied subset of fungi and comprise of species from two distinct phyla: the Ascomycota and the Basidiomycota (Kurtzman and Fell 2006; Suh *et al.* 2006). Similar to other fungi, yeasts exhibit a spectrum of phenotypic heterogeneity. For example, the opportunistic pathogen *Candida albicans* exhibits different types of heterogeneity (Marr *et al.* 2001; Sherry *et al.* 2014). Under standard laboratory conditions, these fungi exist in their yeast form. However, when subjected to conditions that mimic the mammalian host environment (temperature, pH, presence of serum, and so on), they undergo a dramatic transformation where a subset of the population switches their morphogenetic states from yeast cells to long hyphal (tube-like) cells (Noble *et al.* 2017; Kornitzer 2019). These hyphal cells perform nuanced functions including penetrating the host tissue, invading newer niches, and successfully evading the host immune response (Cheng *et al.* 2012; Dühring *et al.* 2015). Interestingly, biofilms formed by *C. albicans* are comprised of cells in both the yeast as well as the hyphal state, and this heterogeneity is critical for them to successfully infect a host (Gulati and Nobile 2016). Another well-studied type of phenotypic heterogeneity in *C. albicans* arises due to white/opaque switching (Soll 2014). *C. albicans* switch between two distinctive types of cells, white and opaque. Each cell type is heritable for multiple generations and switching occurs without a change in the genetic make-up of the microorganism. Distinct properties exhibited by white and opaque cell types result largely from the differential regulation of ~400 genes (Lohse and Johnson 2009). The metabolic state of cells (Lan *et al.* 2002; Ene *et al.* 2016), biofilm formation (Daniels *et al.* 2006), response to host immunity (Kolotila and Diamond 1990; Geiger *et al.* 2004; Lohse and Johnson 2008), and the ability to undergo mating (Miller and Johnson 2002) all show differences between white and opaque cells.

*Saccharomyces cerevisiae*, the model organism that has illuminated diverse aspects of eukaryotic biology, exhibits well-studied phenotypic heterogeneity like *C. albicans* (Palková and Váchová 2016). Under standard laboratory conditions, *S. cerevisiae* cells propagate as ‘unicellular’ yeasts. However, environmental and nutritional cues, including nitrogen starvation result in cells forming pseudohyphae, and eventually filamentous growth. During pseudohyphal development, *S. cerevisiae* cells become elongated and budding occurs synchronously in a unipolar fashion resulting in the production of chains of cells called pseudohyphae (Gancedo 2001). Some (predominantly haploid) *S. cerevisiae* strains also change their morphology after extended growth in standard laboratory medium and these filamentous cells invade into the solid agar medium on which they are growing (Gimeno *et al.* 1992; Roberts and Fink 1994). Pseudohyphal cells are distinct from yeast cells with respect to gene expression patterns, and multiple signaling pathways are required to elicit this switching in response to nitrogen starvation (Gancedo 2001; Cullen and Sprague 2012).

Protein-based elements of inheritance, such as prions, also drive phenotypic heterogeneity in clonal *S. cerevisiae* populations. Prions are ordered, self-assembled aggregates of proteins that can be inherited by daughter cells in *S. cerevisiae* (Uptain and Lindquist 2002; Wickner *et al.* 2007). When a yeast protein self-aggregates and forms prions, there is reduced normal cellular activity of this protein, and this often results in changes in cellular phenotypes (Halfmann *et al.* 2010, 2012). For example, Ure2 is a nitrogen catabolite repressor and when it is active, shuts down the machinery that allows *S. cerevisiae* to utilize poor nitrogen sources. However, when Ure2 self-aggregates and forms prions called [URE3], *S. cerevisiae* cells constitutively utilize poor nitrogen sources (Wickner 1994; Shorter and Lindquist 2005). Hypothetically, if a population of yeast cells were to be subjected to a sudden change in quality of nitrogen sources (rich to poor), the population that exhibits the [URE3] phenotype would have a better chance to survive this change as it is already primed to utilize poor quality nitrogen sources. Yeast cells spontaneously form prions at a frequency of ∼10^−6^. As a result, at any given time, a sizable population of yeast cells will contain a few [prion^+^] cells exhibiting alternate phenotypes. If the environment is such that the [prion^+^] state is beneficial, these cells would then have a greater chance of surviving and proliferating in that environment. If after a period of time, the environment changes to a state where [prion^+^] no longer confers any advantage or causes a growth disadvantage, those cells that do not have the prion would now have a greater chance of surviving, and their percentage would increase in the overall population. Thus, prions can be bet-hedging devices that allow cells to spontaneously switch between phenotypes in a heritable fashion in fluctuating environments (Tuite 2016). In all these examples, phenotypic heterogeneity increases as specific nutrients become limiting in the environment.

## Metabolic heterogeneity in yeast communities

Perhaps the most fundamental form of phenotypic heterogeneity exhibited by *S. cerevisiae* is observed during their development in response to glucose limitation. Early reports describing this phenomenon showed that colonies of *S. cerevisiae* on low-glucose medium formed structurally complex communities with hallmarks of microbial biofilms, including differential gene expression patterns (Mináriková *et al*. 2001;Reynolds and Fink 2001). Subsequent studies identified transcriptional networks and signaling pathways essential for complex community development in response to glucose limitation (Mináriková *et al.* 2001; Granek and Magwene 2010). Interestingly, mounting evidence suggests that these complex biofilm communities exhibit phenotypic heterogeneity within. For example, studies of structured yeast communities developing in glucose-limited environments, showed two populations of cells within, exhibiting high and low metabolic activity (Cáp *et al.* 2012; Palková and Váchová 2016). These groups of cells show heterogeneity with spatial organization with respect to mitochondrial activity, glycolytic activity, autophagy, and general starvation response (Čáp *et al.* 2015; Váchová and Palková 2018).

This raises a fundamental question: how does this phenotypic (particularly metabolic) heterogeneity arise within this clonal community, where the distinct states retain spatial organization? Emerging evidence suggests that the nature and organization of biochemical networks within these cells can explain how cells in heterogeneous states organize and function within the community.

### Self-organizing biochemical systems enabling metabolic heterogeneity in clonal yeast communities

Recent studies now address how this occurs (Varahan *et al.* 2019, 2020). In yeast communities developing in low-glucose environments, most cells are initially in a gluconeogenic state, which is expected in this environment. This state is maintained by stable transcriptional programs that drive gluconeogenesis and related pathways under glucose-limited conditions. Gluconeogenesis is an unavoidable, essential metabolic process for cells growing in low-glucose environments, where cells use available carbon substrates to synthesize the gluconeogenic precursor, oxaloacetate (OAA). Oxaloacetate is converted into phosphoenolpyruvate (PEP) using the PEP carboxykinase enzyme (Pck1) which represents the first committed step of gluconeogenesis. PEP is sequentially converted into fructose-1,6-bisphosphate predominantly using the same enzymes that are involved in glycolysis (in the reverse direction), and fructose-1,6-phosphate is converted into fructose-6-phosphate using the fructose-1,6-bisphosphatase (Fbp1) enzyme which is also gluconeogenesis specific. Fructose-6-phosphate is then converted into glucose-6-phosphate and this acts as a carbon precursor for the synthesis of complex sugars like trehalose and glycogen (Nelson and Cox 2017). However, as the colony develops, groups of cells with dramatically opposite metabolic states emerge. Surprisingly this new population of cells exhibits high glycolytic, along with high pentose phosphate pathway (PPP) activities, and this is fuelled by the break-down of trehalose obtained from the gluconeogenic cells which is converted into glucose-6-phosphate. This trehalose break-down is simultaneously utilized for the synthesis of ribose sugars using the PPP and sequentially oxidized to pyruvate to meet the energy demands of the cells. Despite the nutrient environment being glucose-depleted, these cells showed all hallmarks of yeast cells in glucose-replete environments (Varahan *et al.* 2019).

**Figure 1:**
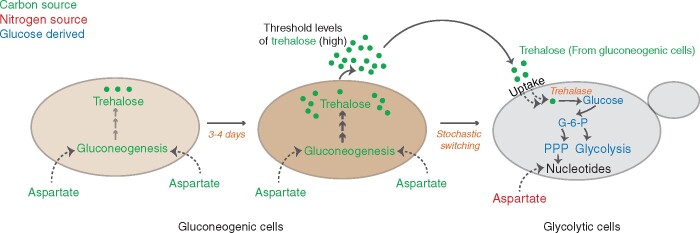
Emergence of metabolic heterogeneity in a clonal group of cells growing in glucose-limited conditions. Cells in glucose-limited environments perform gluconeogenesis. This is primarily fuelled by aspartate (nonlimiting resource) which acts as a carbon source. As the community develops, gluconeogenic reserves build-up and trehalose concentration in the extracellular environment increases. At a threshold concentration of extracellular trehalose, some cells stochastically switch to a high glycolytic, PPP state. This state is fuelled by the utilization of trehalose (as a carbon source), and aspartate (nonlimiting) is primarily used as a nitrogen source for the biosynthesis of nucleotides. This consumption of trehalose by the glycolytic cells results in decreased concentrations of external trehalose, bringing the concentration below the threshold. This in turn restricts the other, remaining cells in their original gluconeogenic state and they continue to synthesize trehalose. This results in a self-organized community that exhibits specialization of function and division of labor.

The explanation for the appearance and maintenance of these glycolytic cells came from emergent, self-organizing biochemical principles. Initially, within a colony, cells were highly gluconeogenic, and an outcome of gluconeogenesis is the production and accumulation of an originally limiting resource, the disaccharide trehalose - which is made of two glucose molecules. As trehalose amounts increase, this newly available resource becomes abundantly available to all cells. Some cells (likely stochastically) increase trehalose uptake and switch to consuming and breaking-down trehalose for glucose, and this results in global, stable transcriptional changes which allow them to sustain high rates of glycolysis (Varahan *et al.* 2019). As these cells switch to glycolysis and consume more trehalose, the amounts of available trehalose start depleting. Hence, the remaining cells can no longer switch, and remain trehalose ‘producers’ that continue in a gluconeogenic state (Figure 1). Simulated coarse-grained resource consumption models can remarkably recapitulate this phenomenon, at the level of both patterns forming as well as the organization of specialized cell groups within the colony (Varahan *et al.* 2019). In effect, this emergence of metabolic heterogeneity and organization of the population of cells with distinct metabolic states can be driven by a self-organizing biochemical network of gluconeogenic cells producing a resource and glycolytic cells consuming it. The outcome of this system is what appears to be a fully functional cross-feeding system within a clonal colony, with cells in one state (gluconeogenic) sustaining the cells in the other (glycolytic) (Figure 1).

## How such specialization leads to metabolic division of labor?

For reproductive success and survival, cellular communities must perform multiple complex biological tasks. The metabolic burden on any single cell can be large if it is required to carry out all necessary biochemical reactions to enable growth and manage survival alone, and this can lead to fitness trade-offs. Therefore, a strategy deployed by many cell communities to overcome this problem is to break down complex processes into discrete sets of steps and divide tasks among different individuals of the community (Giri *et al.* 2019). Division of labor enhances the efficiency of biological processes that are executed within communities by eliminating the need to perform or switch between multiple tasks, leading to fitness advantages (van Gestel *et al.* 2015; West and Cooper 2016). Due to this, division of labor is widely prevalent across microbial communities and is found at different levels of biological organization (Michod 2007; Tarnita *et al.* 2013).

A key parameter dictating the success of any cellular community is the effective utilization of available nutrients. Depending on the type and quality of nutrients available, cells within communities often switch between metabolic states, to adapt to fluctuating availability of nutrients (Johnson *et al.* 2012; Campbell *et al.* 2015). However, some biochemical reactions are mutually incompatible, *i.e.*, the same cell cannot perform these reactions simultaneously. For example, mass-action driven metabolic fluxes ensure that the same cell cannot sustain high rates of glycolysis and gluconeogenesis at the same time. Therefore, dividing metabolic labor can become a strategy that allows microbial communities to survive fluctuating nutrient environments (Tsoi *et al.* 2018). Another classic example comes from cyanobacterial communities. Cyanobacterial species *Synechococcus elongatus* requires photosynthesis and nitrogen fixation for its survival and growth. However, these two biochemical processes are mutually incompatible, due to distinct oxygen requirements. Therefore, cells in these cyanobacterial communities segregate into photosynthetic cells and nitrogen-fixing cells and this metabolic division of labor allows these communities to perform both essential tasks simultaneously (Flores and Herrero 2010; Rossetti *et al.* 2010). Metabolic division of labor has typically been observed in mixed microbial communities. For example, the nitrification process in the soil exhibits metabolic division of labor wherein the ammonia-oxidizing bacteria convert the ammonia into nitrite and nitrite-oxidizing bacteria convert nitrite to nitrate (Beeckman *et al.* 2018). Cross-feeding observed in mixed microbial populations is another example of metabolic division of labor since each species within is responsible for producing distinct metabolites that are shared amongst the different members of the community (Schink 2002; Wintermute and Silver 2010).

### Metabolic cross-feeding systems

**Figure 2:**
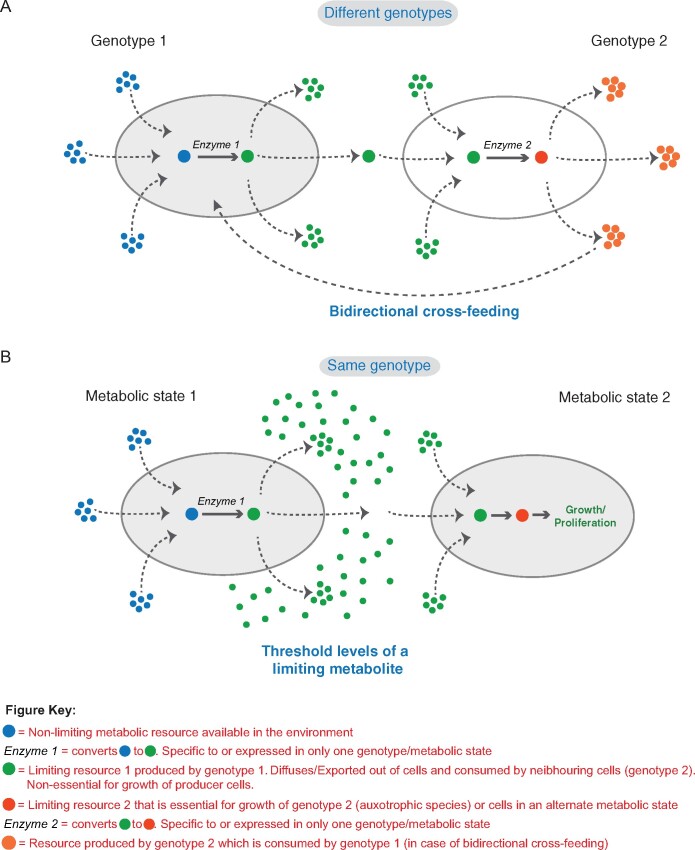
Metabolic cross-feeding in microbial communities, either of different genotypes, or clonal. (A) Complex microbial communities are comprised of different species with distinct genotypes. In dual-species communities, a particular species within (genotype 1) consumes the nonlimiting resource available in the external environment and produces a limiting resource necessary for the growth of the second species (genotype 2). In specific scenarios, a second species can produce a limiting resource necessary for the growth of the other species in these communities. Complementary auxotrophies result in metabolic syntrophy and allows the community as a whole to flourish. (B) In clonal communities, when the levels of a limiting resource produced from nonlimiting resources reach a threshold concentration, it can result in the emergence of cells in a new metabolic state (which consumes this limiting resource to maintain this state). This switch to an alternate metabolic state is reversible and depends on the availability of the limiting resource.

In many naturally occurring microbial communities, the challenging task of breaking down very complex nutrients available in an external environment is performed collectively by multiple species. Molecules resulting from the metabolism within one strain are metabolized by other strains, and this phenomenon is called cross-feeding (Figure 2A). Cross-feeding has been explored in many heterogeneous microbial communities comprised of either different species of microbes in single community or laboratory-engineered cell populations where metabolic interdependencies are generated via genetic manipulation that create nutrient auxotrophies (Schink 2002; Shou *et al.* 2007; Wintermute and Silver 2010; Mee *et al.* 2014; Campbell *et al.* 2015). These studies reveal how complex communities can achieve metabolic heterogeneity when each genotype (typically an auxotroph for a nutrient) divides labor to produce or receive a specific metabolite. This sharing of metabolic resources enables increased growth and fitness of the community. Cross-feeding systems typically consist of specialist strains that performs a restricted group of biochemical tasks and relies on another species for obtaining metabolites and other products (auxotrophies) needed for their growth (Figure 2A). Generally, species that complement each other’s auxotrophies become an interdependent community. The resulting metabolic syntrophy allows the community as a whole to flourish in a given environment (Morris *et al.* 2013; Pande *et al.* 2014). Individuals in a cross-feeding community can have higher overall fitness compared to a community made of a single species of a generalist microbe that can perform all the biochemical tasks on its own. This growth advantage comes from the division of metabolic labor wherein a fitness cost of producing a resource needed for the growth of the complementary auxotroph is less than the benefit of not having to produce other resources when they are provided by their partner in the cross-feeding system (Axelrod and Hamilton 1981; Hillesland and Stahl 2010).

Interestingly, even clonal communities of microorganisms exhibit metabolic division of labor. For example, a recent study showed that a clonal population of *Bacillus subtilis* exhibit metabolic division of labor wherein a subpopulation of cells produce acetate which allows these bacteria to grow rapidly. But to mitigate the toxicity of acetate at higher concentrations, a distinct subpopulation of cells in these clonal communities start converting the acetate to acetoin (which is nontoxic) and this allows the community as a whole to grow in a detoxified environment (Rosenthal *et al.* 2018). In work in yeast, a general principle emerged of how threshold levels of specific metabolites drive self-organizing biochemical networks enabling the emergence of metabolic heterogeneity and subsequent metabolic division of labor in clonal yeast communities (Varahan *et al.* 2019, 2020).

### Resource threshold-dependent metabolic cross-feeding within yeast communities

**Figure 3:**
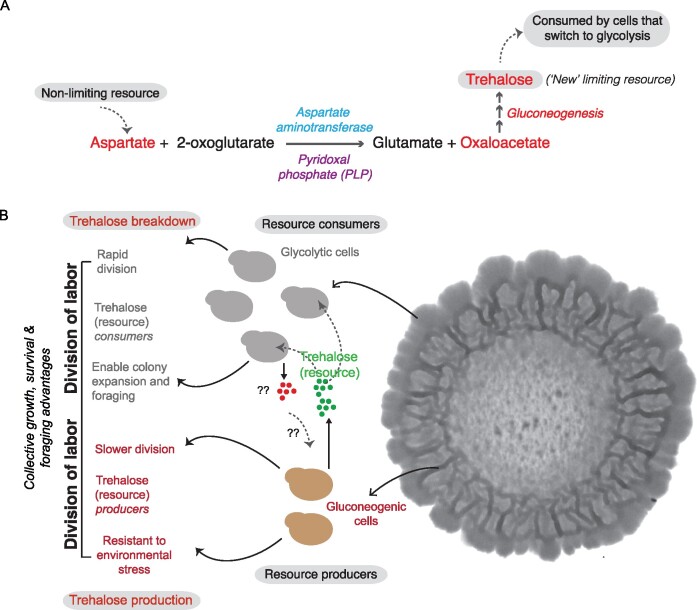
Biochemically versatile metabolites orchestrate the emergence and maintenance of metabolic heterogeneity resulting in co-operative division of labor in yeast. (A) Aspartate (nonlimiting resource) acts as a carbon substrate to produce the gluconeogenic precursor, oxaloacetate via a transamination reaction. Oxaloacetate is converted into trehalose (limiting resource) via gluconeogenesis and this results in the emergence of the glycolytic sub-population of cells that consumes this resource to fuel their metabolic state. (B) Cooperative division of labor exhibited by the gluconeogenic and glycolytic cells provides collective growth, survival, and foraging advantages to the community as a whole. Gluconeogenic cells accumulate trehalose and this protects them from various environmental stresses like desiccation, freeze/thaw cycles, and temperature fluctuations that cells in yeast communities often face in their natural environments. The glycolytic cells (that consume the trehalose) allow yeast colonies to rapidly expand over time, forage, and reach new territory. The glycolytic cells are ideally positioned spatially to allow the colony to expand and forage toward new territory. It is possible that the glycolytic cells produce a metabolic resource that is consumed by the gluconeogenic cells to maintain their metabolic state.

Strikingly, as introduced earlier, in clonal yeast communities, cells self-organize into effective cross-feeding, mutualistic groups within a community. In glucose-limited conditions, the prevalent metabolic pathway (gluconeogenesis) leads to the production and accumulation of trehalose. This new (formerly limiting) resource allows some cells to stochastically switch to a trehalose-consuming state, which is glycolytic, and these glycolytic cells effectively function as auxotrophs (for trehalose) (Varahan *et al.* 2019). The glycolytic cells obtain glucose via the uptake and breakdown of this trehalose, which they obtain from the gluconeogenic cells (Figure 1). This is remarkably efficient as a cross-feeding system. Importantly, this self-organized biochemical system that leads to a cross-feeding network is enabled by the build-up of a novel resource (trehalose) to sufficiently high levels (a “threshold”), which did not exist initially in the environment (Figure 2B). This leads to a deeper question, of how the high flux through gluconeogenesis which produces the resource (trehalose) itself is sustained. Underlying this finding was that the entire cross-feeding system and trehalose production is sustained by existing, nonlimiting resources of specific amino acids (Varahan *et al.* 2020). These relatively nonlimiting amino acids (primarily aspartate) act as carbon sources in gluconeogenic cells to drive gluconeogenesis (Figure 3A). But they also have alternate fates that are metabolically important in sustaining the cells that switch to a glycolytic state as well (Figure 1; Varahan *et al.* 2020).

This highlights the concept of biochemically versatile metabolites and how their metabolic flexibility can drive, enable, or control biochemical networks that set up cross-feeding systems of specialized cell states in clonal cell communities.

## How cellular metabolic states and biochemically versatile metabolites orchestrate the emergence and maintenance of metabolic heterogeneity?

Cellular metabolism obviously enables many biological processes that are critical for the functioning of cells. It is now apparent that certain metabolites directly control cell growth and proliferation, functioning both as signaling molecules as well as molecules that can be utilized for different anabolic/catabolic processes. These include metabolites like ATP, NAD(P)H, and acetyl-CoA (Westheimer 1987; Cantó *et al.* 2015; Pietrocola *et al.* 2015). Other studies demonstrate that metabolites like s-adenosylmethionine (SAM), carbamoyl phosphate, and UDP-glucose also act as sentinel metabolites and drive biochemical pathways required for growth and proliferation (Walsh *et al.* 2018). These metabolites allow cells to efficiently perform methyl transfers (SAM), phosphoryl transfers (ATP), electron and ADP-ribosyl transfers (NAD(P)H/NAD(P)^+^), acyl transfers (acetyl-CoA and carbamoyl phosphate), and glucosyl transfers (UDP-glucose), which in turn drive multiple transcriptional and metabolic transformations across primary biochemical pathways (Nelson and Cox 2017; Walsh *et al.* 2018). Depending on how much of these metabolites are present, they can direct overall metabolic programs toward growth, differentiation, or survival states.

However, there are also other metabolites, with biochemically versatile roles, that work “behind the scenes” to enable metabolically distinct states to sustain themselves. These molecules can have multiple uses, and may (depending on how they are utilized) change the availability of some of the sentinel metabolites described above. Studies from the organized, metabolically specialized cell groups within yeast colonies illustrate how this might be so. Here, the two molecules central to setting up the cross-feeding system are the disaccharide trehalose, and the amino acid aspartate (Figure 1; Varahan *et al.* 2019, 2020). Both molecules can function differently and drive completely distinct metabolic pathways depending on the metabolic state of the cells. When trehalose (which is produced by gluconeogenesis during glucose starvation) is consumed and broken down into glucose, it can fuel high rates of glycolysis and the PPP, as it does in the highly glycolytic cells within the colonies growing in glucose-limited conditions (Varahan *et al.* 2019). Indeed, the roles of trehalose as a critical requirement for cells to exit quiescence and enter rapidly dividing states are well documented (Silljé *et al.* 1999; Futcher 2006; Shi *et al.* 2010; Shi and Tu 2013). Yet, trehalose also has distinct, critical functions in the gluconeogenic cells, or cells entering quiescence. In these cells, it behaves as a membrane and protein protectant molecule, allowing them to survive multiple freeze/thaw cycles and extreme desiccation (Wiemken 1990; D’Amore *et al.* 1991; Shi *et al.* 2010; Erkut *et al.* 2011, 2016). This plasticity in its use itself therefore can directly determine cell state. Interestingly, in this gluconeogenic/glycolytic cell cross-feeding system observed in yeast, trehalose is initially a limiting resource that builds up to above a threshold, when it triggers some cells to switch to a consumer state (Varahan *et al.* 2019). The production of this resource is itself driven by a more abundant, and more metabolically versatile resource, the amino acid aspartate. In glucose-limited conditions, aspartate primarily functions as a carbon precursor for driving gluconeogenesis (Vengayil *et al.* 2019; Varahan *et al.* 2020). Aspartate is easily converted to the gluconeogenic precursor oxaloacetate, and therefore drives high flux through gluconeogenesis. This is sufficient to produce sufficient trehalose required to reach the levels that can then trigger a switch of some cells to a glycolytic (trehalose-consuming) state (Figure 3A; Varahan *et al.* 2019, 2020). Interestingly, aspartate can be important in glycolytic cells, which have solved their requirements of carbon for rapid growth, for very different reasons. The glucose from trehalose drives glycolysis and the PPP, leading to the production of ribose sugars required for nucleotide synthesis. But in order to make sufficient nucleotides, cells require aspartate to function as a *nitrogen* donor, and aspartate therefore plays a critical, distinct role in sustaining the glycolytic cell state (Figure 1; Varahan *et al.* 2020). Thus, this metabolic flexibility of trehalose and aspartate allows the existence of the glycolytic cell state in these communities while these metabolites simultaneously are essential for the maintenance of the gluconeogenic state.

What these studies describe are cross-feeding systems that are largely formed by self-organizing biochemical networks, which themselves have metabolically flexible molecules as the driving entities behind them. What this more broadly entails is that while sentinel metabolites power cell metabolism (Walsh *et al.* 2018), for the emergence of interdependent, specialized groups of cells within clonal communities, such metabolically flexible, versatile molecules play central roles. Identifying and understanding the spectrum of such metabolites and how they can enable organized phenotypic heterogeneity in clonal cell communities would be an exciting area of future study. Here, the many roles and fates of amino acids, and their ability to drive distinct arms of carbon and nitrogen metabolism (Amelio *et al.* 2014; Yang and Vousden 2016; Walvekar *et al.* 2018; Yoo *et al.* 2020), are likely to become increasingly apparent.

## Implications of metabolic specialization and division of labor in clonal yeast communities

How might the separation of biochemical processes and division of labor occur within spatially restricted clonal yeast communities be advantageous to the community, and might this inform the roles of such systems in other microbes? For example, this begs the question, why should the cells growing in low-glucose conditions perform high amounts of glycolysis, when they can survive via gluconeogenesis? Interestingly, by maintaining cells in two metabolic states, with a spatial organization, a host of advantages can be conferred to the community as a whole (Figure 3B). Several studies have shown that cells use their metabolism to counter a variety of stresses that they encounter in their environments. For example, in response to oxidative stress, cells divert their metabolic flux from glycolysis toward the PPP to increase cytoplasmic NADPH, which provides the redox power for known antioxidant systems (Pollak *et al.* 2007; Ralser *et al.* 2007), or counter oxidative insults by harvesting lysine that is available in their extracellular environment. As a consequence, NADPH which would otherwise be utilized for the biosynthesis of lysine is channelled toward the synthesis of glutathione, which allows cells to counter the oxidative insults efficiently (Olin-Sandoval *et al.* 2019). In low-glucose environments, the gluconeogenic cells are very well adapted to survive environmental insults that yeast communities often face in natural environments like (a) desiccation, (b) freeze/thaw cycles, and (c) temperature fluctuations (Erkut *et al.* 2016; Varahan *et al.* 2020). This comes from the accumulation of trehalose, which as described earlier is a biochemical endpoint metabolite of gluconeogenesis, and is a versatile protectant (Wiemken 1990; D’Amore *et al.* 1991). Contrastingly, the emergent glycolytic cells provide new advantages to cells within the colony. These cells (which are themselves sustained by the gluconeogenic cells) proliferate rapidly, and allow the colonies to expand over time, forage, and reach new territory. The glycolytic cells are ideally positioned spatially (at the periphery of the colony) to allow the colony to expand and forage toward new territory (Varahan *et al.* 2020), but themselves cannot exist without the gluconeogenic cells. The ability to forage for nutrients is a key survival strategy deployed by many microbes growing in nutrient-limiting conditions, and thus this becomes possible within the yeast colonies only due to the division of labor that sustains distinct metabolic states (Figure 3B).

## Possible evolutionary implications of the metabolic division of labor: sympatric speciation?

Metabolic specialization within a community confers growth and fitness advantages to the community as a whole. However, it is tempting to speculate on the possibility of a longer-term trajectory. When the environmental selection pressure (nutrient limitations) that drives metabolic heterogeneity in isogenic communities is sustained over very long periods (which yeast and most microbes experience in the wild), it could potentially lead to mutagenic events where subsets of the clonal population locked into a particular metabolic state evolve auxotrophies and subsequently perform restricted biochemical tasks. Such auxotrophs will exist only as long as they are supported by the complementary population that forms the cross-feeding system. This is of course seen in other syntrophic microbial populations which have lost their ability to facultatively switch back to any alternate metabolic state. In other words, could prolonged metabolic heterogeneity as a consequence of constant environmental selective pressure therefore be imagined as an evolutionary precursor of sympatric speciation events in isogenic communities?

Speciation is the evolution of a new species from a surviving ancestral species (Wu 2001; Mallet 2008; Shapiro *et al.* 2016). Multiple factors contribute to speciation events and these include (a) gradual evolution of genetic incompatibilities, (b) specialization to an ecological niche, and (c) alterations to the chromosome via homologous recombination or horizontal gene transfer events (Presgraves 2010; Shapiro and Polz 2015; Shapiro *et al.* 2016). In yeasts, hybridization events have led to speciation by combining genomes that have evolved independently. These genomes, when combined, provide advantages to the hybrid individuals. New hybrids become a different species only when they are self-fertile and exhibit reproductive isolation from their parental strains (Greig *et al.* 2002; Dettman *et al.* 2007; Leducq *et al.* 2016). Several examples of the emergence of new species of yeast as a consequence of hybridization exist. A classic example is *Saccharomyces pastorianus*, the source of lager beer, which is a result of a hybridization event between *Saccharomyces cerevisiae* and *Saccharomyces eubayanus*. This newly formed hybrid inherited properties from both parental strains, making it optimal for lager beer brewing (Libkind *et al.* 2011; van den Broek *et al.* 2015; Gorter De Vries *et al.* 2019).

However, hybridization may also dilute beneficial properties that exist in parental strains. This is particularly true when sympatric speciation is ecologically driven, and occurs in order to adapt to an environmental niche. This phenomenon is called hybrid dysfunction, wherein species that have evolved to adapt to a particular environmental niche (environment-specific adaptations) produce hybrids with reduced fitness in either parental habitat (Johnson 2008, 2010). We can apply this concept of ecologically driven hybrid dysfunction to clonal yeast colonies with division of labor and make plausible speculations. The metabolic heterogeneity in clonal yeast communities is an ecologically driven event in response to glucose limitation. Potentially, when this clonal community of yeast remains in glucose-limited conditions for extended periods of time, the two metabolically distinct population of cells could evolve into two populations that have diminished ability to switch back to an alternate metabolic state, and rather adapt to grow exclusively in a specific environment (gluconeogenic or glycolytic). Because the gluconeogenic and glycolytic cells exhibit diametrically opposite metabolic states, they would exhibit hybrid dysfunction or hybrid incompatibility as it is impossible to have a cell performing glycolysis and gluconeogenesis at the same time. Indeed, very recent studies suggest that this might be possible. In yeast continuously propagated in melibiose as a carbon source, which can break down to galactose and glucose, the release of these as a public good might allow adaptive diversification of a clonal population (Mahilkar *et al.* 2021).

It, therefore, is a plausible speculation that metabolic heterogeneity that occurs in isogenic communities as a consequence of sustained selective pressure may result in ecologically driven speciation events. Could multispecies communities that exhibit metabolic cross-feeding have originally started as isogenic groups of cells that carried out metabolic division of labor as an adaptation strategy to a specific environmental niche?
